# Maternal Prenatal Infections and Biliary Atresia in Offspring

**DOI:** 10.1001/jamanetworkopen.2023.50044

**Published:** 2024-01-03

**Authors:** Wei-Hao Wang, Fang-Yu Chiu, Tzu-Tung Kuo, Yu-Hsuan Joni Shao

**Affiliations:** 1 Graduate Institute of Biomedical Informatics, College of Medical Science and Technology, Taipei Medical University, Taipei, Taiwan; 2 Department of Pediatrics, Changhua Christian Hospital, Changhua, Taiwan; 3 Department of Obstetrics and Gynecology and Women’s Health, Taichung Veterans General Hospital, Taichung, Taiwan; 4 Department of Obstetrics and Gynecology, Lienchiang County Hospital, Lienchiang, Taiwan; 5 Health Data Analytics and Statistics Center, Office of Data Science, Taipei Medical University, Taipei, Taiwan; 6 Clinical Big Data Research Center, Taipei Medical University Hospital, Taipei, Taiwan

## Abstract

**Importance:**

Investigations into the association of antepartum maternal infections with the pathogenesis of biliary atresia (BA) in human offspring are insufficient.

**Objective:**

To examine the association between prenatal infections in mothers and the development of BA in their offspring.

**Design, Setting, and Participants:**

This population-based case-control study obtained administrative data from the Taiwan National Health Insurance Research Database with linkage to the Taiwan Maternal and Child Health Database, capturing demographic and medical information on nearly all 23 million of the Taiwan population. The cohort comprised 2 905 978 singleton live births among mother-infant dyads between January 1, 2004, and December 31, 2020, in Taiwan. The case group of infants with BA was identified from use of *International Classification of Diseases* diagnostic codes for BA and subsequent Kasai procedure or liver transplant. The control group was randomly selected from infants without BA, representing approximately 1 in 1000 study population. Data analyses were performed from May 1 to October 31, 2023.

**Exposure:**

Prenatal maternal infections, including intestinal infection, influenza, upper airway infection, pneumonia, soft-tissue infection, and genitourinary tract infection.

**Main Outcomes and Measures:**

The main outcome was exposure to prenatal maternal infections. Inverse probability weighting analysis was performed by building a logistic regression model to estimate the probability of the exposure observed for a particular infant and using the estimated probability as a weight in subsequent analyses. The weighted odds ratio (OR) estimated by logistic regressions was then used to assess the risk of BA in offspring after prenatal maternal infections.

**Results:**

Among the mother-infant dyads included, 447 infants with BA were cases (232 females [51.9%]) and 2912 infants without BA were controls (1514 males [52.0%]). The mean (SD) maternal age at childbirth was 30.7 (4.9) years. Offspring exposed to prenatal intestinal infection (weighted OR, 1.46; 95% CI, 1.17-1.82) and genitourinary tract infection (weighted OR, 1.22; 95% CI, 1.05-1.41) in mothers exhibited a significantly higher risk of BA. Furthermore, maternal intestinal infection (weighted OR, 6.05; 95% CI, 3.80-9.63) and genitourinary tract infection (weighted OR, 1.55; 95% CI, 1.13-2.11) that occurred during the third trimester were associated with an increased risk of BA in offspring.

**Conclusions and Relevance:**

Results of this case-control study indicate an association between prenatal intestinal infection and genitourinary tract infection in mothers and BA occurrence in their offspring. Further studies are warranted to explore the underlying mechanisms of this association.

## Key Points

**Question:**

Is there an association between antepartum maternal infectious diseases and biliary atresia (BA) in offspring?

**Findings:**

In this case-control study of 447 infants with a BA diagnosis and 2912 controls, offspring born to mothers with prenatal intestinal infection and genitourinary tract infection had a significantly higher risk of developing BA compared with offspring who were not exposed to such maternal infections.

**Meaning:**

The findings of this study suggest that the underlying mechanism of the association between antepartum maternal genitourinary tract infection and offspring BA occurrence warrants further exploration.

## Teaser

This case-control study examines the association between prenatal exposure to intestinal infection, influenza, upper airway infection, pneumonia, soft-tissue infection, and genitourinary tract infection in mothers during pregnancy and the development of biliary atresia in their offspring.

## Introduction

Biliary atresia (BA) is a rare yet devastating hepatobiliary disease that is the primary cause for pediatric liver transplant.^1^ Although substantial research has been conducted, the underlying etiology of BA remains elusive. According to current theories, the principal pathomechanism of BA involves inflammation and fibrosis of hepatocytes and cholangiocytes,^2^ particularly after exposure to viral infections during the early neonatal period.^3^ However, studies have shown that BA may begin in utero.^4^ The association of maternal infections during pregnancy with development of BA in offspring is less understood.

Although no conclusive evidence currently demonstrates a direct association between maternal bacterial colonization and the fetus,^5^ studies revealed that maternal commensal microbiota still participate in the development of the fetal immune system through bacteria-derived metabolites or endobiotics.^6,7^ In mice, alternation in maternal intestinal microorganisms during gestation may be a factor in inflammation and T-cell overactivation in offspring, which is characteristic of BA.^8,9,10^ Furthermore, maternal microbial signatures were found to be associated with the susceptibility of the offspring mice to BA.^11^

In humans, microbiotic dysbiosis, which refers to a disruption in the balanced microbiota composition, often occurs after viral or bacterial infections as well as systemic antibiotic administration.^12,13,14^ This condition is associated with tissue inflammation and immune dysregulation.^15,16,17^ Therefore, we hypothesized that maternal infection diseases during the prenatal period are associated with the occurrence of BA in offspring. Accordingly, we conducted a population-based study to examine the association between prenatal infections in mothers and the development of BA in their offspring.

## Methods

### Data Source

This case-control study used data derived from the Taiwan National Health Insurance Research Database (NHIRD) and the Taiwan Maternal and Child Health Database (MCHD).^18,19^ The National Health Insurance covers nearly all 23 million Taiwanese citizens, and the NHIRD contains comprehensive health information for all of the beneficiaries, including their identification (ID) number, demographic characteristics, diagnoses and prescriptions from each medical visit, and medical examinations and surgical procedures.^18^ In the MCHD, IDs for Taiwanese offspring born after 2004 and their parents are stored along with perinatal characteristics, including gestational age, birth weight, delivery method, and number of pregnancies.^19,20^ The Taiwan Health and Welfare Data Science Center maintained and provided these databases for medical research in a deidentified manner.^18,20^ The Taipei Medical University–Joint Institutional Review Board approved the study and waived the informed consent requirement because deidentified data were used. We followed the Strengthening the Reporting of Observational Studies in Epidemiology (STROBE) reporting guideline.^21^

### Study Cohort

The Taiwan Health and Welfare Data Science Center used the same algorithm to encrypt private information in the NHIRD and the MCHD so that 1 individual had an identical encrypted ID in these 2 data sets. We linked the NHIRD and MCHD by encrypted IDs^19^ to create a birth cohort consisting of all singleton live births in Taiwan between January 1, 2004, and December 31, 2020, with medical claims data. We retrieved a total of 2 905 978 mother-infant dyads from the data set.

In the initial identification of candidates for the case group, we used a criterion of at least 3 outpatient claims or 1 inpatient claim with *International Classification of Diseases, Ninth Revision (ICD-9)* diagnostic code 751.61 or *International Statistical Classification of Diseases and Related Health Problems, Tenth Revision (ICD-10)* diagnostic code Q44.2 for BA. Recognizing that some offspring with prolonged jaundice might receive a tentative diagnosis of BA during medical visits but may not ultimately have the condition, we further verified the presence of BA by confirming a subsequent Kasai procedure or liver transplant before 6 months of age. This criterion was chosen because surgical biliary-enteric drainage and liver transplant were the only 2 curative therapies for BA, and nearly all affected offspring, including preterm infants, received surgical intervention within 180 days after birth.^22,23,24^

The control group consisted of randomly selected infants without a BA diagnosis, representing approximately 1 in 1000 study population, born each year from January 1, 2004, to December 31, 2020. The control group was matched to the case group on the basis of year of birth.

### Exposure and Covariates

The exposure of interest was maternal infectious diseases during pregnancy, including intestinal infection, genitourinary tract infection, influenza, upper airway infection, pneumonia, and soft-tissue infection.^25,26,27,28^ We defined these infections according to relevant *ICD-9* or *ICD-10* diagnostic codes used for at least 1 outpatient or inpatient claim in the NHIRD.^18^ eTable 1 in Supplement 1 provides the relevant *ICD-9* and *ICD-10* diagnostic codes used to identify these diseases.

Because intrauterine biliary development varies at different stages of gestation,^29^ we also documented the trimester during which the maternal infection occurred: preconception (3 months before conception), first trimester (before the 12th week of gestation), second trimester (13th to 28th week of gestation), and third trimester (after the 29th week of gestation). The date of conception was calculated by subtracting the gestational age from the date of birth.

Taking into account the potential role of family socioeconomic status in the occurrence of infectious diseases during gestation,^30^ we grouped each family according to their annual insurance premium, using the high-, middle-, and low-income tertiles to represent their socioeconomic level. We used the annual insurance premium as a proxy for socioeconomic level because Taiwan’s National Health Insurance program operates under a progressive insurance rate system; that is, people with higher income pay higher annual insurance premiums.^31^ We also obtained additional perinatal confounders of BA and maternal infection: female sex, preterm labor (childbirth before 37 weeks’ gestation), infant birth weight, preeclampsia, and maternal diabetes.^24,32,33^

### Statistical Analysis

We calculated the birth prevalence of BA during the study period. We built a logistic regression model to estimate the probability (propensity score) of exposure to prenatal maternal infections for every infant in the cohort according to the given baseline demographic characteristics.^34^ Subsequently, we used these propensity scores to calculate the weights of each infant to construct pseudopopulations with a balanced covariate distribution between exposed and unexposed groups. The standardized mean difference was used to compare the difference in means between groups in units of SD before and after inverse probability weighting. Using the weighted data, we performed logistic regression analyses to determine the weighted odds ratio (OR) of the occurrence of BA in offspring exposed to each type of prenatal maternal infection compared with nonexposed offspring.^34^ Considering that the severity of infection or administration of systemic antibiotics might alter offspring outcomes, we performed sensitivity analyses to extract maternal infections that necessitated inpatient treatment or systemic antibiotics and evaluated their association with BA risk in offspring. The prescribed antibiotics were recognized by the Anatomical Therapeutic Chemical Classification System code recorded in the NHIRD.

The χ2 test was used to assess the distributional differences in categorical variables between the case and control groups. Two-sided *P* < .05 was considered statistical significance. All statistical analyses were performed from May 1 to October 31, 2023, using SAS, version 9.4 (SAS Institute Inc).

## Results

### Demographic Characteristics

From among the mother-infant dyads in the data set from January 1, 2004, to December 31, 2020, we included 447 singleton infants with BA as the case group (215 males [48.1%], 232 females [51.9%]) and randomly selected 2912 infants without BA as the control group (1514 males [52.0%], 1398 females [48.0%]) (Table 1; Figure). The mean (SD) birth weight of the infants was 3078 (443) g, and the male to female ratio was 1.06. The mean (SD) maternal age at childbirth was 30.7 (4.9) years. The annual birth prevalence of BA was 1.54 per 10 000 live births over the study period (eTable 2 in Supplement 1). Table 1 provides the demographic characteristics of the mother-infant dyads in both the case and control groups. We found that BA was more likely to affect preterm (<37 weeks’ gestation) offspring in the case group compared with the control group (51 [11.4%] vs 220 [7.6%]; *P* = .005 by χ^2^ test). Infant sex, maternal age, maternal diabetes, preeclampsia, and family socioeconomic level had no association with the development of BA. The standardized mean differences of covariates after inverse probability weighting were all less than 0.1, indicating balanced covariate distribution^35^ (Table 1).

**Table 1.** Demographic Characteristics of the Mother-Infant Dyads

Characteristic	Group, No. (%)		SMD	
	Case (n = 447)	Control (n = 2912)	Before weighting	After weighting
Infant sex				
Male	215 (48.1)	1514 (52.0)	0.0342	0.0052
Female	232 (51.9)	1398 (48.0)	0.0342	0.0052
Maternal age at childbirth, mean (SD), y	30.5 (5.01)	30.8 (4.86)	0.2104	0.0000
Infant birth weight, mean (SD), g	2949 (457)	3098 (451)	0.0214	0.0006
Gestational age at birth, wk				
<37	51 (11.4)	220 (7.6)^a^	0.0299	0.0007
≥37	396 (88.6)	2692 (92.5)^a^	0.0299	0.0007
Delivery method				
Vaginal	301 (67.3)	1880 (64.6)	0.0794	0.0002
Cesarean	146 (32.7)	1032 (35.4)	0.0794	0.0002
Maternal diabetes				
Yes	35 (7.8)	262 (9.0)	0.0522	0.0014
No	412 (92.2)	265 (91.0)	0.0522	0.0014
Pregestational diabetes				
Yes	11 (2.5)	76 (2.6)	0.0267	0.0013
No	436 (97.5)	2836 (97.4)	0.0267	0.0013
Gestational diabetes				
Yes	25 (5.6)	209 (7.2)	0.0458	0.0020
No	422 (94.4)	2703 (92.8)	0.0458	0.0020
Preeclampsia				
Yes	9 (2.0)	28 (1.0)	0.0214	0.0019
No	438 (98.0)	2884 (99.0)	0.0214	0.0019
Eclampsia				
Yes	0	0	NA	NA
No	447 (100)	2912 (100)	NA	NA
Family socioeconomic level				
Low income	155 (34.7)	922 (31.7)	0.1389	0.0193
Middle income	153 (34.2)	987 (33.9)	0.0522	0.0418
High income	139 (31.1)	1003 (34.4)	0.1927	0.0230

Abbreviations: NA, not applicable; SMD, standardized mean difference.

^a^ The sum was greater than 100% due to rounding.

**Figure.**
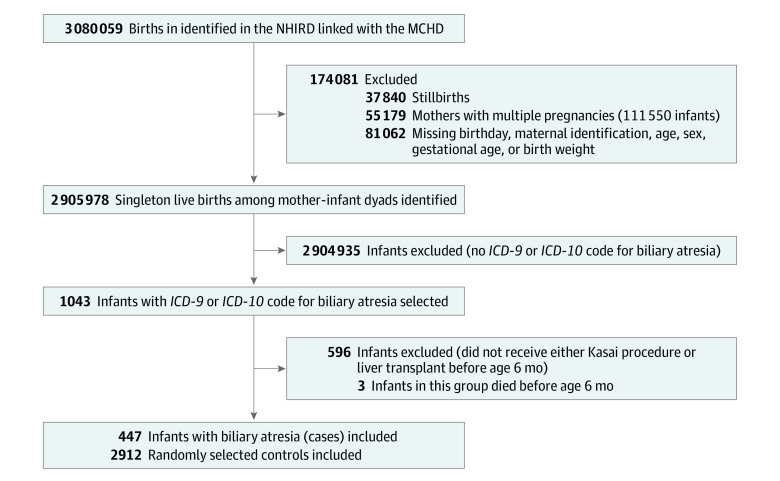
Flow of the Mother-Infant Dyads *ICD-9* indicates *International Classification of Diseases, Ninth Revision*; *ICD-10*, *International Statistical Classification of Diseases and Related Health Problems, Tenth Revision*; MCHD, the Maternal and Child Health Database; and NHIRD, National Health Insurance Research Database.

### Maternal Infections

We identified 3803 episodes of prenatal maternal infections. In both the case and control groups, upper airway infection was the most common disease (325 [72.7%] and 2081 [71.5%], respectively), followed by genitourinary tract infection, intestinal infection, influenza, soft-tissue infection, and pneumonia (Table 2). Among all infectious diseases diagnosed during preconception or pregnancy, intestinal infections (weighted OR, 1.46; 95% CI, 1.17-1.82) and genitourinary tract infections (weighted OR, 1.22; 95% CI, 1.05-1.41) were associated with a significantly increased risk of BA in offspring (Table 2). The weighted ORs were obtained from propensity score–weighted logistic regression that considered variables such as maternal age, gestational age, infant sex, infant birth weight, delivery method, maternal diabetes, preeclampsia, eclampsia, and family socioeconomic level.

**Table 2.** Maternal Infection Between Case and Control Groups

	Group, No. (%)		OR (95% CI)	Weighted OR (95% CI)^a^
	Case (n = 447)	Control (n = 2912)		
Intestinal infection				
No	410 (9.7)	2752 (94.5)	1 [Reference]	1 [Reference]
Yes	37 (8.3)	160 (5.5)	1.55 (1.07-2.25)	1.46 (1.17-1.82)
Influenza				
No	410 (91.7)	2712 (93.1)	1 [Reference]	1 [Reference]
Yes	37 (8.3)	200 (6.9)	1.22 (0.85-1.76)	1.18 (0.95-1.47)
Upper airway infection				
No	122 (17.3)	831 (28.5)	1 [Reference]	1 [Reference]
Yes	325 (72.7)	2081 (71.5)	1.00 (0.78-1.27)	1.02 (0.86-1.21)
Pneumonia				
No	442 (98.9)	2886 (99.1)	1 [Reference]	1 [Reference]
Yes	5 (1.1)	26 (0.9)	1.26 (0.44-3.84)	1.26 (0.67-2.37)
Soft-tissue infection				
No	409 (91.5)	2708 (93.0)	1 [Reference]	1 [Reference]
Yes	38 (8.5)	204 (7.0)	1.23 (0.86-1.77)	1.15 (0.92-1.43)
Genitourinary tract infection				
No	343 (76.7)	2326 (79.9)	1 [Reference]	1 [Reference]
Yes	104 (23.3)	586 (20.1)	1.27 (0.95-1.53)	1.22 (1.05-1.41)

Abbreviation: OR, odds ratio.

^a^ Weighted ORs were estimated using a propensity score–weighted logistic regression that considered the following variables that were imbalanced between infected and noninfected populations: maternal age, gestational age, infant sex, infant birth weight, delivery method, maternal diabetes, preeclampsia, and family socioeconomic level.

Thirty-seven cases had a history of prenatal intestinal infection, including 9 (2.0%) with bacterial infection, 4 (0.9%) with viral infection, fewer than 3 patients with fungal infection, and 23 (5.2%) with infection by a nonspecific pathogen. No intestinal parasitic infection was found (Table 3). Offspring born to mothers with antepartum viral enterocolitis were at a significantly higher risk of BA (weighted OR, 2.18; 95% CI, 1.19-4.00). Although intestinal infection was associated with a high percentage of nonspecific pathogens, we still found a significantly increased risk of BA in offspring within this group (weighted OR, 1.43; 95% CI, 1.10-1.85).

**Table 3.** Pathogen of Intestinal Infection Between Case and Control Groups

	Group, No. (%)		OR (95% CI)	Weighted OR (95% CI)^a^
	Case (n = 447)	Control (n = 2912)		
Bacterial gastroenteritis				
No	438 (97.8)	2859 (98.2)	1 [Reference]	1 [Reference]
Yes	9 (2.0)	53 (1.8)	1.58 (0.59-4.23)	1.04 (0.71-1.52)
Viral gastroenteritis				
No	443 (99.1)	2901 (99.6)	1 [Reference]	1 [Reference]
Yes	4 (0.9)	11 (0.4)	2.38 (0.76-7.51)	2.18 (1.19-4.00)
Parasite infection				
No	447 (100)	2912 (100)	1 [Reference]	1 [Reference]
Yes	0	0	NA	NA
Fungal infection				
No	>444^b^	>2909^b^	1 [Reference]	1 [Reference]
Yes	<3^b^	<3^b^	6.53 (0.41-104.54)	6.61 (1.28-34.22)
Others (nonspecific pathogen)				
No	424 (94.9)	2814 (96.6)	1 [Reference]	1 [Reference]
Yes	23 (5.2)	98 (3.4)	1.56 (0.98-2.48)	1.43 (1.10-1.85)

Abbreviations: NA, not applicable; OR, odds ratio.

^a^ Weighted ORs were estimated using a propensity score–weighted logistic regression that considered the following variables that were imbalanced between infected and noninfected populations: maternal age, gestational age, infant sex, infant birth weight, delivery method, maternal diabetes, preeclampsia, and family socioeconomic level.

^b^ Under National Health Insurance Research Database regulations, cells with a value of 1 or 2 must be relabeled as <3 to safeguard confidentiality.

When we assessed the outcome of maternal infection during different stages of pregnancy, we observed a significantly increased risk of BA in offspring associated with intestinal infection and genitourinary tract infection during the third trimester. Specifically, the weighted OR for intestinal infection was 6.05 (95% CI, 3.80-9.63), and the weighted OR for genitourinary tract infection was 1.55 (95% CI, 1.13-2.11) (Table 4).

**Table 4.** Trimesters of Maternal Infection Between Case and Control Groups

	Group, No. (%)		OR (95% CI)	Weighted OR (95% CI)^a^
	Case (n = 447)	Control (n = 2912)		
**Intestinal infection**				
During preconception: 3 mo before pregnancy				
No	439 (98.2)	2478 (98.4)	1 [Reference]	1 [Reference]
Yes	8 (1.8)	40 (1.6)	1.02 (0.48-2.17)	0.96 (0.64-1.44)
During the first trimester				
No	444 (99.3)	2881 (98.9)	1 [Reference]	1 [Reference]
Yes	3 (0.7)	31 (1.1)	0.63 (0.19-2.07)	0.56 (0.30-1.08)
During the second trimester				
No	440 (98.4)	2880 (98.9)	1 [Reference]	1 [Reference]
Yes	7 (1.6)	32 (1.1)	1.43 (0.63-3.27)	1.40 (0.90-2.19)
During the third trimester				
No	437 (97.8)	2902 (99.7)	1 [Reference]	1 [Reference]
Yes	10 (2.2)	10 (0.3)	6.64 (2.75-16.05)	6.05 (3.80-9.63)
**Genitourinary tract infection**				
During preconception: 3 mo before pregnancy				
No	432 (96.6)	2787 (95.7)	1 [Reference]	1 [Reference]
Yes	15 (3.4)	125 (4.3)	0.77 (0.45-1.34)	0.75 (0.54-1.03)
During the first trimester				
No	436 (95.7)	2826 (97.1)	1 [Reference]	1 [Reference]
Yes	11 (2.5)	86 (3.0)	0.83 (0.44-1.57)	0.84 (0.59-1.21)
During the second trimester				
No	434 (97.1)	2790 (95.8)	1 [Reference]	1 [Reference]
Yes	13 (2.9)	122 (4.2)	0.69 (0.38-1.23)	0.66 (0.47-0.94)
During the third trimester				
No	430 (96.2)	2840 (97.5)	1 [Reference]	1 [Reference]
Yes	17 (3.8)	72 (2.5)	1.56 (0.91-2.67)	1.55 (1.13-2.11)

Abbreviation: OR, odds ratio.

^a^ Weighted ORs were estimated using a propensity score–weighted logistic regression that considered the following variables that were imbalanced between infected and noninfected populations: maternal age, gestational age, infant sex, infant birth weight, delivery method, maternal diabetes, preeclampsia, and family socioeconomic level.

### Sensitivity Analyses and Effect Modification

Prenatal maternal intestinal infection, whether treated in inpatient (weighted OR, 1.48; 95% CI, 1.02-2.14) or outpatient (weighted OR, 1.45; 95% CI, 1.11-1.90) departments, was associated with an increased risk of BA in offspring. For genitourinary tract infection, only severe cases requiring hospitalization were associated with BA risk in offspring (weighted OR, 1.81; 95% CI, 1.46-2.25). Offspring of mothers who were hospitalized for other prenatal infections did not show an elevated risk of developing BA (eTable 3 in Supplement 1). However, offspring of mothers who received antibiotics to treat influenza, upper airway infection, soft-tissue infection, and genitourinary tract infection had an increased risk of developing BA (eTable 4 in Supplement 1). For example, the weighted OR for antibiotics-treated genitourinary tract infection was 1.27 (95% CI, 1.07-1.52).

eTables 5 and 6 in Supplement 1 present the analyses of effect modification. We found a more pronounced increase in the risks for BA after exposure to prenatal maternal intestinal infection (weighted OR, 1.75 [95% CI, 1.29-2.36] vs 1.20 [95% CI, 0.86-1.68]) and genitourinary tract infection (weighted OR, 1.33 [95% CI, 1.08-1.63] vs 1.09 [95% CI, 0.88-1.36]) among female offspring compared with male offspring. However, this outcome was observed in full-term offspring. Late-preterm infants (gestational age >32 weeks but <37 weeks) did not exhibit significant change in risk. The number of extremely preterm infants in this cohort was limited.

## Discussion

This population-based case-control study revealed that prenatal maternal intestinal infection and genitourinary tract infection during the third trimester were associated with a higher risk of BA in offspring. This observation suggests that antepartum maternal infections might play a potential role in the pathogenesis of BA, warranting further investigation.

The results showed that prematurity was associated with BA, which is consistent with findings of previous reports.^24,32^ One possible assumption is that an intrauterine event, such as maternal infection, exerts stress on the fetus, leads to preterm birth, and is associated with the development of BA. It is also possible that having a premature immune system, along with dysregulated responses to infection or toxins, is a factor in progressive inflammation in the hepatobiliary system.^24^ Antepartum infectious diseases have been associated with preterm labor,^28^ which introduces the possibility of confounding bias in this study. To address this concern, we implemented the inverse probability weighting approach to balance the different distribution of potential confounders such as infant sex, gestational age, infant birth weight, and other maternal infectious diseases.^34^ The findings suggest that prenatal maternal gut and genitourinary tract infection are independent risk factors for BA in offspring.

Emerging evidence indicates that the maternal gut and genitourinary tract microbiota play a role in the growth of the gastrointestinal and immune systems in offspring.^36,37,38^ Dysbiosis frequently arises after infections and has been associated with several human diseases, particularly inflammatory diseases.^12,14,15,16,17^ Theoretically, the pathogenic microorganisms in the maternal body cannot directly invade the fetus due to the presence of the blood-placenta barrier.^6^ However, the metabolites of the disrupted maternal microbiota after maternal infectious diseases could be transferred to the fetus via the placenta and subsequently induce tissue inflammation, including in the hepatobiliary tract of the developing fetus.^6,12,17^ In the sensitivity analysis, we observed an association between antibiotic use during pregnancy and increased risk of BA in offspring (eTable 4 in Supplement 1). This result was conceivable and was associated with the main finding since antibiotics can disturb the natural balance of gut bacteria, leading to dysbiosis.^14^ In particular, offspring of mothers who had bacterial intestinal infections or were treated with antibiotics did not show an increased risk of BA. This finding could be due to the relatively small sample size of the cohort or the adverse effects of antibiotics being mitigated by the benefits from inhibiting the growth of deleterious bacteria.

In human fetal hepatobiliary embryonic development, the primitive intrahepatic bile duct epithelium cells begin to proliferate in the 12th week of gestation and to form tubular structures in the 20th week of gestation.^29^ The maturation of the biliary system commences during early embryonic development and is subject to explicit cell-signaling regulation, and excessive cytokine secretion might play a role in hepatic and biliary tissue inflammation.^29,39,40^ In a mouse model, maternal infections induce T-cell activation and trigger excessive interleukin 6 production, leading to increased susceptibility to gut inflammation and biliary duct injury in the offspring.^7^ Lipopolysaccharide in the membranes of pathogenic enteric bacteria (eg, *Salmonella*) can also provoke secretion of oxidative agents, resulting in liver damage.^41^ These rationales might help to explain the findings of this study. However, after examining maternal infections during different gestational stages, a genitourinary tract infection occurring in the second trimester demonstrated protective properties against BA in offspring, as indicated by weighted logistic regression. The crude OR did not show statistical significance (Table 4). Further research is needed for a more in-depth investigation.

### Strengths and Limitations

One of the primary strengths of this study was the use of population-based administrative data, which could substantially reduce the potential for recall bias or selection bias.^42^ Another strength was the study’s large-scale approach, particularly in investigating the rare disease of BA within a population-based setting. Additionally, we used national mother-infant linked data to investigate the association of maternal factors with offspring risk. To our knowledge, this study is the first to reveal a possible association between maternal infections and the development of BA in human offspring.

The study also has several limitations. First, we identified the case group by *ICD-9* and *ICD-10* diagnostic codes and after Kasai operation or liver transplant. While the accuracy of this algorithm has not been validated, it is noteworthy that the case numbers and estimated prevalence of BA at birth in this study aligned with a previous study using the Taiwan registry data.^43^ Second, because the NHIRD and the MCHD did not contain the results of laboratory tests, we identified maternal infectious diseases by using only *ICD-9* and *ICD-10* diagnostic codes rather than by directly detecting pathogens in stool samples or body fluids. The accuracy of this approach also has not been validated. Third, we lacked information on other risk factors for BA, such as exposure to environmental toxins and genetic predispositions.^1^ Although rare, the inclusion of an offspring with Alagille syndrome in the case group was possible given that the Kasai procedure and liver transplant were treatment options for cholestasis.^44^ Fourth, fewer than 5% of infants with BA had a history of maternal infection. The findings could explain only a small proportion of BA occurrence. Fifth, the number of offspring with BA who were exposed to prenatal maternal pneumonia was low. Risk estimation in this group should be inspected with caution due to the potential bias for sparse data.^45^ Sixth, the common pathogens of infectious diarrhea vary by geographic regions,^46^ and different dietary habits and lifestyles also have a distinct role in the composition of the gut microbiota.^47,48^ The applicability of the present study to other populations requires external validation.

## Conclusions

This case-control study observed an association between prenatal intestinal infection and genitourinary tract infection in mothers and BA occurrence in their offspring, the exact underlying mechanism for which warrants further exploration. The findings also indicate the importance of additional BA surveillance in offspring of pregnant women with these diseases.

## Notes

Supplement 1. **eTable 1.** The *International Classification of Diseases (ICD)* Codes for Disease Identification **eTable 2.** The Annual Birth Prevalence of Biliary Atresia During Study Period **eTable 3.** Odds Ratios for Biliary Atresia in Offspring: Comparison of Maternal Prenatal Infection Treated in Outpatient and In-Patient Departments **eTable 4.** Odds Ratios for Biliary Atresia in Offspring: Comparison of Maternal Prenatal Infection Treated With and Without Antibiotics Usage **eTable 5.** The Impact of Prenatal Maternal Infections on Offspring Biliary Atresia Risk in Male and Female Infants **eTable 6.** The Impact of Prenatal Maternal Infections on Offspring Biliary Atresia Risk in Infants Born at Different Gestational Ages

Supplement 2.
Data Sharing Statement
