# Supplementary material for: Maternal Prenatal Infections and Biliary Atresia in Offspring
Source: JAMA Netw Open. 2024 Jan 3;7(1):e2350044. doi: 10.1001/jamanetworkopen.2023.50044 (PMC10765264; doi:10.1001/jamanetworkopen.2023.50044)
Supplement: Supplement 1. — eTable 1. The International Classification of Diseases (ICD) Codes for Disease Identification eTable 2. The Annual Birth Prevalence of Biliary Atresia During Study Period eTable 3. Odds Ratios for Biliary Atresia in Offspring: Comparison of Maternal Prenatal Infection Treated in Outpatient and In-Patient Departments eTable 4. Odds Ratios for Biliary Atresia in Offspring: Comparison of Maternal Prenatal Infection Treated With and Without Antibiotics Usage eTable 5. The Impact of Prenatal Maternal Infections on Offspring Biliary Atresia Risk in Male and Female Infants eTable 6. The Impact of Prenatal Maternal Infections on Offspring Biliary Atresia Risk in Infants Born at Different Gestational Ages [file jamanetwopen-e2350044-s001.pdf]

Supplemental Online Content

Wang WH, Chiu FY, Kuo TT, Shao YHJ. Maternal prenatal infections and biliary atresia in offspring. *JAMA Netw Open*. 2024;7(1):e2350044. doi:10.1001/jamanetworkopen.2023.50044

eTable 1. The *International Classification of Diseases* (*ICD*) Codes for Disease Identification

eTable 2. The Annual Birth Prevalence of Biliary Atresia During Study Period

eTable 3. Odds Ratios for Biliary Atresia in Offspring: Comparison of Maternal Prenatal Infection Treated in Outpatient and In-Patient Departments

eTable 4. Odds Ratios for Biliary Atresia in Offspring: Comparison of Maternal Prenatal Infection Treated With and Without Antibiotics Usage

eTable 5. The Impact of Prenatal Maternal Infections on Offspring Biliary Atresia Risk in Male and Female Infants

eTable 6. The Impact of Prenatal Maternal Infections on Offspring Biliary Atresia Risk in Infants Born at Different Gestational Ages

This supplemental material has been provided by the authors to give readers additional information about their work.

© 2024 Wang WH et al. *JAMA Network Open*

eTable 1. The *International Classification of Diseases* (*ICD*) codes for disease identification.

ICD-9	description	ICD-10	description
**Intestinal infection**			
001.0-009.3	Intestinal infectious diseases	A00-A09	Intestinal infectious diseases
041.86	Helicobacter pylori	B37.0	Candidal stomatitis
	Candidiasis of mouth	B96.81	Helicobacter pylori [H. pylori] as the cause of diseases classified elsewhere
112.0			
112.84	Candidal esophagitis	B37.81	Candidal esophagitis
112.85	Candidal enteritis	B37.82	Candidal enteritis
540-543	Appendicitis	B46.2	Gastrointestinal mucormycosis
562	Diverticula of intestine	K35-K37	Appendicitis
		K57	Diverticular disease
		B97.5	Reovirus as the cause of diseases classified elsewhere
**Central nervous system infection**			
047	Meningitis due to enterovirus	A83	Mosquito-borne viral encephalitis
048	Other enterovirus diseases of central nervous system	A84	Tick-borne viral encephalitis
049	Other non-arthropod-borne viral diseases of central nervous system	A85	Other viral encephalitis, not elsewhere classified
052.2	Postvaricella myelitis	A86	Unspecified viral encephalitis
053.0	Herpes zoster with meningitis	A87	Viral meningitis
053.1	Herpes zoster with unspecified nervous system complication	A88	Other viral infections of central nervous system, not elsewhere classified
054.72	Herpes simplex meningitis	A89	Unspecified viral infection of central nervous system
054.74	Herpes simplex myelitis	B00.3	Herpesviral meningitis
055.0	Postmeasles encephalitis	B00.4	Herpesviral encephalitis
056.0	Rubella; with neurological complications	B01.0	Varicella meningitis
058.2	Other human herpesvirus encephalitis	B01.1	Varicella encephalitis, myelitis and encephalomyelitis
062	Mosquito-borne viral encephalitis	B02.0	Zoster encephalitis
063	Tick-borne viral encephalitis	B02.1	Zoster meningitis
064	Viral encephalitis transmitted by other and unspecified arthropods	B05.0	Measles complicated by encephalitis
066.41	West Nile Fever with encephalitis	B05.1	Measles complicated by meningitis
066.42	West Nile Fever with other neurologic manifestation	B06.01	Rubella encephalitis
072.1	Mumps meningitis	B06.02	Rubella meningitis
072.2	Mumps encephalitis	B08	Other viral infections characterized by skin and mucous membrane lesions, not elsewhere classified
112.83	Candidal meningitis	B10.0	Other human herpesviruses
130.0	Meningoencephalitis due to toxoplasmosis	B26.1	Mumps meningitis
320	Bacterial meningitis	B26.2	Mumps encephalitis
321	Meningitis due to other organisms	B37.5	Candidal meningitis

© 2024 Wang WH et al. *JAMA Network Open*

322	Meningitis of unspecified cause	B38.4	Coccidioidomycosis meningitis
323	Encephalitis, myelitis, and encephalomyelitis	B43.1	Pheomycotic brain abscess
324	Intracranial and intraspinal abscess	B58.2	Toxoplasma meningoencephalitis
		G00	Bacterial meningitis, not elsewhere classified
		G01	Meningitis in bacterial diseases classified elsewhere
		G02	Meningitis in other infectious and parasitic diseases classified elsewhere
		G06	Intracranial and intraspinal abscess and granuloma
		G07	Intracranial and intraspinal abscess and granuloma in diseases classified elsewhere
**Upper airway infection**			
460-466	ACUTE RESPIRATORY INFECTIONS	J00-J06	Acute upper respiratory infections
**Pneumonia**			
052.1	Varicella (hemorrhagic) pneumonitis	B25.0	Cytomegaloviral pneumonitis
055.1	Postmeasles pneumonia	B37.1	Pulmonary candidiasis
073.0	Ornithosis with pneumonia	B38.0~ B38.2	Pulmonary coccidioidomycosis
112.4	Candidiasis of lung	B39.0 ~ B39.2	Pulmonary histoplasmosis capsulati
114.0, 114.4, 114.5	Pulmonary coccidioidomycosis	B40.0~ B40.3	pulmonary blastomycosis
130.4	Pneumonitis due to toxoplasmosis	B41.0	Pulmonary paracoccidioidomycosis
480-486	Pneumonia	B42.0	Pulmonary sporotrichosis
		B44.0, B44.0	Pulmonary aspergillosis
		B45.0	Pulmonary cryptococcosis
		B46.0	Pulmonary mucormycosis
		J12-J18	Pneumonia
**Influenza**			
487, 488	Influenza	J09-J11	Influenza
**Cardiovascular infection**			
074.2	Coxsackie carditis	B26.82	Mumps myocarditis
112.81	Candidal endocarditis	B37.6	Candidal endocarditis
130.3	Myocarditis due to toxoplasmosis	B33.2	Viral carditis
114.2	Coccidioidal meningitis	B40.81	Blastomycotic meningoencephalitis
		B42.81	Cerebral sporotrichosis
		B45.1	Cerebral cryptococcosis
		B46.1	Rhinocerebral mucormycosis
		B58.81	Toxoplasma myocarditis
		B58.82	Toxoplasma myositis
		I40.0	Infective myocarditis
**Genitourinary tract infection**			
112.1, 112.2	Candidiasis of urogenital sites	B37.3, B37.4	Candidiasis of urogenital sites
590	Infections of kidney	N10	Acute pyelonephritis

© 2024 Wang WH et al. *JAMA Network Open*

599.0	Urinary tract infection, site not specified	N39.0	Urinary tract infection, site not specified
646.5	Asymptomatic bacteriuria in pregnancy	O23	Infections of genitourinary tract in pregnancy
646.6	Infections of genitourinary tract in pregnancy		
**Soft tissue infection**			
110	Dermatophytosis	B35	Dermatophytosis
111	Dermatomycosis other and unspecified	B36	Other superficial mycoses
112.3	Candidiasis of skin and nails	B37.2	Candidiasis of skin and nail
680-686	Infections of skin and subcutaneous tissue	B38.3	Cutaneous coccidioidomycosis
		B40.3	Cutaneous blastomycosis
		B43.0	Cutaneous chromomycosis
		B43.2	Subcutaneous pheomycotic abscess and cyst
		B45.2	Cutaneous cryptococcosis
		B46.3	Cutaneous mucormycosis
		L00-L08	Infections of the skin and subcutaneous tissue
**Other infection**			
050	Smallpox	A51	Early syphilis
051	Cowpox and paravaccinia	A80	Acute poliomyelitis
052 (except 052.1, 52.2)	Chickenpox	A81	Atypical virus infections of central nervous system
053 (except 053.0, 53.1)	Herpes zoster	A82	Rabies
054 (except 054.7, 054.74)	Herpes simplex	A90-A99	Arthropod-borne viral fevers and viral hemorrhagic fevers
055 (except 055.0, 055.1)	Measles	B00 (except B00.3, B00.4)	Herpesviral [herpes simplex] infections
056 (except 056.0)	Rubella	B01(except B01.0, B01.1)	Varicella
057	Other viral exanthemata	B02(except B02.0, B02.1)	Zoster [herpes zoster]
058 (except 058.2)	Other human herpesvirus	B04	Monkeypox
059	Other poxvirus infections	B05 (except B05.0, B05.1)	Measles
060	Yellow fever	B06 (except B06.01, B06.02)	Rubella
061	Dengue	B25 (except B25.0)	Cytomegaloviral disease
065	Arthropod-borne hemorrhagic fever	B26.0 (except B26.1, B26.2, B26.82)	Mumps
066 (except 066.41, 066.42)	Other arthropod-borne viral diseases	B27	Infectious mononucleosis
071	Rabies	B33 (except B33.2)	Other viral diseases, not elsewhere classified
072 (except 072.1, 072.2)	Mumps	B37.7	Candidal sepsis
073 (except 073.0)	Ornithosis	B37.89	Other sites of candidiasis
074 (except 074.2)	Specific diseases due to Coxsackie virus	B37.9	Candidiasis, unspecified
075	Infectious mononucleosis	B38.7~B38.9	Coccidioidomycosis
076	Trachoma	B39.3~B39.5, B39.9	Histoplasmosis
077	Other diseases of conjunctiva due to viruses and Chlamydiae	B40.89	Other forms of blastomycosis

© 2024 Wang WH et al. *JAMA Network Open*

078	Other diseases due to viruses and Chlamydiae	B40.9	Blastomycosis, unspecified
079	Viral and chlamydial infection in conditions classified elsewhere and of unspecified site	B41.7~ B41..9	Paracoccidioidomycosis
090-099	Syphilis and other venereal diseases	B42.7, B42.8	Sporotrichosis
112.5	Disseminated candidiasis	B43.8, B43.9	Chromomycosis, unspecified
112.89	Other candidiasis of other specified sites	B44.7, B44.8	Aspergillosis
112.9	Candidiasis of unspecified site	B45.7~ B45.9	Cryptococcosis
130 (except 130.0, 130.3, 130.4)	Toxoplasmosis	B46.4, B46.5	Mucormycosis
114.1, 114.3, 114.9	Coccidioidomycosis	B46.8	Other zygomycoses
116	Blastomycotic infection	B46.9	Zygomycosis, unspecified
117, 118	Other mycoses	B47	Mycetoma
		B48	Other mycoses, not elsewhere classified
		B49	Unspecified mycosis
		B58 (except B58.2, B58.81, B58.82)	Toxoplasmosis
		B97 (except B97.5)	Viral agents as the cause of diseases classified elsewhere
		O98.1	Syphilis complicating pregnancy, childbirth and the puerperium
**Maternal comorbidity**			
250	Diabetes mellitus	E08-E13	Diabetes mellitus
648.0	Diabetes mellitus in the mother, but complicating pregnancy, childbirth, or the puerperium	O24	Diabetes mellitus in pregnancy, childbirth, and the puerperium
642.4	Mild or unspecified pre-eclampsia	O11	Pre-existing hypertension with pre-eclampsia
642.5	Severe pre-eclampsia	O14	Pre-eclampsia
642.6	Eclampsia	O15	Eclampsia

© 2024 Wang WH et al. *JAMA Network Open*

eTable 2. The annual birth prevalence of biliary atresia during study period

Year	Number of singleton livebirths	Number of cases with biliary atresia	Birth prevalence
2004	182705	37	2.03
2005	175223	32	1.83
2006	174504	30	1.72
2007	175733	23	1.31
2008	171988	23	1.34
2009	169859	24	1.41
2010	136926	25	1.83
2011	166989	29	1.74
2012	188817	27	1.43
2013	174928	24	1.37
2014	180114	29	1.61
2015	192345	27	1.40
2016	187033	32	1.71
2017	174865	20	1.14
2018	161239	20	1.24
2019	155714	19	1.22
2020	136549	26	1.90
Overall	2905531	447	1.54

© 2024 Wang WH et al. *JAMA Network Open*

eTable 3. Odds ratios for biliary atresia in offspring: comparison of maternal prenatal infection treated in outpatient and in-patient departments

	Case, n (%)	Control, n (%)	OR (95% CI)	wOR (95% CI)[a]
Intestinal infection				
No	410 (91.7)	2752 (94.5)	1 (Reference)	1 (Reference)
Yes	37 (8.3)	160 (5.5)	1.55 (1.07-2.25)	1.46 (1.17-1.82)
Need in-patient care	13 (2.9)	54 (1.9)	1.62 (0.87-2.99)	1.48 (1.02-2.14)
Out-patient care only	24 (5.4)	106 (3.6)	1.52 (0.96-2.40)	1.45 (1.11-1.90)
Influenza infection				
No	410 (91.7)	2712 (93.1)	1 (Reference)	1 (Reference)
Yes	37 (8.3)	200 (6.9)	1.22 (0.85-1.76)	1.18 (0.95-1.47)
Need in-patient care	<3[b]	13	1.02 (0.23-4.53)	1.04 (0.43-2.52)
Out-patient care only	>34[b]	187	1.24 (0.85-1.80)	1.19 (0.95-1.49)
Upper airway infection				
No	122 (27.3)	831 (28.5)	1 (Reference)	1 (Reference)
Yes	325 (72.7)	2081 (71.5)	1.06 (0.85-1.33)	1.10 (0.94-1.29)
Need in-patient care	11 (2.5)	62 (2.1)	1.21 (0.62-2.36)	1.11 (0.70-1.74)
Out-patient care only	314 (70.3)	2019 (69.3)	1.06 (0.85-1.33)	1.10 (0.94-1.29)
Pneumonia				
No	442 (98.9)	2886 (99.1)	1 (Reference)	1 (Reference)
Yes	5 (1.1)	26 (0.9)	1.26 (0.48-3.29)	1.24 (0.71-2.15)
Need in-patient care	>2[b]	18	1.45 (0.49-4.31)	1.34 (0.70-2.58)
Out-patient care only	<3[b]	8	0.82 (0.10-6.54)	1.03 (0.37-2.89)
Soft tissue infection				
No	409 (91.5)	2708 (93.0)	1 (Reference)	1 (Reference)
Yes	38 (8.5)	204 (7.0)	1.23 (0.86-1.77)	1.15 (0.92-1.43)
Need in-patient care	6 (1.3)	25 (0.9)	1.59 (0.65-3.90)	1.43 (0.83-2.46)
Out-patient care only	32 (7.2)	179 (6.2)	1.18 (0.80-1.75)	1.11 (0.88-1.40)
Genitourinary tract infection				
No	343 (76.7)	2326 (79.9)	1 (Reference)	1 (Reference)
Yes	104 (23.3)	586 (20.1)	1.20 (0.95-1.53)	1.22 (1.05-1.41)
Need in-patient care	42 (9.4)	158 (5.4)	1.80 (1.26-2.58)	1.81 (1.46-2.25)
Out-patient care only	62 (13.9)	428 (14.7)	0.98 (0.74-1.31)	1.00 (0.84-1.19)

[a] Weighted odds ratios (wOR) were estimated by using a propensity score weighted logistic regression considering the following variables that were imbalanced between infected and non-infected population: mother's age, gestational age, infant sex, birth weight, deliver method, maternal diabetes, pre-eclampsia, and socioeconomic level.

© 2024 Wang WH et al. *JAMA Network Open*

[b] Under NHIRD regulations, cells with a value of 1 or 2 are to be relabeled as '<3' to safeguard confidentiality.

© 2024 Wang WH et al. *JAMA Network Open*

eTable 4. Odds ratios for biliary atresia in offspring: comparison of maternal prenatal infection treated with and without antibiotics usage

	Case, *n* (%)	Control, *n* (%)	OR (95% CI)	wOR (95% CI)[a]
Intestinal infection				
No	410 (91.7)	2752 (94.5)	1 (Reference)	1 (Reference)
Yes	37 (8.3)	160 (5.5)	1.55 (1.07-2.25)	1.46 (1.17-1.82)
Treat with antibiotics	14 (3.1)	64 (2.2)	1.47 (0.82-2.64)	1.32 (0.93-1.89)
Treat without antibiotics	23 (5.2)	96 (3.3)	1.61 (1.01-2.57)	1.55 (1.18-2.04)
Influenza infection				
No	410 (91.7)	2712 (93.1)	1 (Reference)	1 (Reference)
Yes	37 (8.3)	200 (6.9)	1.22 (0.85-1.76)	1.18 (0.95-1.47)
Treat with antibiotics	19 (4.3)	73 (2.5)	1.72 (1.03-2.88)	1.72 (1.27-2.33)
Treat without antibiotics	18 (4)	127 (4.4)	0.94 (0.57-1.55)	0.89 (0.66-1.19)
Upper airway infection				
No	122 (27.3)	831 (28.5)	1 (Reference)	1 (Reference)
Yes	325 (72.7)	2081 (71.5)	1.06 (0.85-1.33)	1.10 (0.94-1.29)
Treat with antibiotics	130 (29.1)	750 (25.8)	1.18 (0.91-1.54)	1.24 (1.03-1.49)
Treat without antibiotics	195 (43.6)	1331 (45.7)	1.00 (0.78-1.27)	1.02 (0.86-1.21)
Pneumonia				
No	442 (98.9)	2886 (99.1)	1 (Reference)	1 (Reference)
Yes	5 (1.1)	26 (0.9)	1.26 (0.48-3.29)	1.24 (0.71-2.15)
Treat with antibiotics	>2[b]	20	1.31 (0.44-3.84)	1.26 (0.67-2.37)
Treat without antibiotics	<3[b]	6	1.09 (0.13-9.06)	1.17 (0.37-3.65)
Soft tissue infection				
No	409 (91.5)	2708 (93.0)	1 (Reference)	1 (Reference)
Yes	38 (8.5)	204 (7.0)	1.23 (0.86-1.77)	1.15 (0.92-1.43)
Treat with antibiotics	23 (5.2)	100 (3.4)	1.52 (0.96-2.42)	1.45 (1.10-1.92)
Treat without antibiotics	15 (3.4)	104 (3.6)	0.96 (0.55-1.66)	0.88 (0.63-1.21)
Genitourinary tract infection				
No	343 (76.7)	2326 (79.9)	1 (Reference)	1 (Reference)
Yes	104 (23.3)	586 (20.1)	1.20 (0.95-1.53)	1.22 (1.05-1.41)
Treat with antibiotics	65 (14.5)	347 (11.9)	1.27 (0.95-1.70)	1.27 (1.07-1.52)
Treat without antibiotics	39 (8.7)	239 (8.2)	1.11 (0.77-1.58)	1.13 (0.92-1.40)

[a] Weighted odds ratios (wOR) were estimated by using a propensity score weighted logistic regression considering the following variables that were

© 2024 Wang WH et al. *JAMA Network Open*

imbalanced between infected and non-infected population: mother's age, gestational age, infant sex, birth weight, deliver method, maternal diabetes, pre-eclampsia, and socioeconomic level.

[b] Under NHIRD regulations, cells with a value of 1 or 2 are to be relabeled as '<3' to safeguard confidentiality.

© 2024 Wang WH et al. *JAMA Network Open*

eTable 5. The impact of prenatal maternal infections on offspring biliary atresia risk in male and female infants.

	wOR (95% CI)[a]
Baby sex: male	
With versus without prenatal maternal intestinal infection	1.20 (0.86-1.68)
With versus without prenatal maternal genitourinary tract infection	1.09 (0.88-1.36)
Baby sex: female	
With versus without prenatal maternal intestinal infection	1.75 (1.29-2.36)
With versus without prenatal maternal genitourinary tract infection	1.33 (1.08-1.63)

[a] Weighted odds ratios (wOR) were estimated by using a propensity score weighted logistic regression considering the following variables that were imbalanced between infected and non-infected population: mother's age, socioeconomic level, gestational age, birth weight, deliver method, and maternal diabetes, and pre-eclampsia.

© 2024 Wang WH et al. *JAMA Network Open*

eTable 6. The impact of prenatal maternal infections on offspring biliary atresia risk in infants born at different gestational ages.

	wOR (95% CI)[a]
Gestational age: <32 weeks and 0 day (extremely and very preterm)	
With versus without prenatal maternal intestinal infection	NA (no case)
With versus without prenatal maternal genitourinary tract infection	6.57 (1.01-42.89)
Gestational age: 32 weeks and 0 day to < 37 weeks and 0 day (late preterm)	
With versus without prenatal maternal intestinal infection	1.74 (0.81-3.72)
With versus without prenatal maternal genitourinary tract infection	1.21 (0.77-1.91)
Gestational age: more than 37 weeks and 0 day (full term)	
With versus without prenatal maternal intestinal infection	1.48 (1.17-1.87)
With versus without prenatal maternal genitourinary tract infection	1.20 (1.02-1.40)

[a] Weighted odds ratios (wOR) were estimated by using a propensity score weighted logistic regression considering the following variables that were imbalanced between infected and non-infected population: mother's age, socioeconomic level, sex, birth weight, deliver method, and maternal diabetes, and pre-eclampsia.

© 2024 Wang WH et al. *JAMA Network Open*
