# Supplementary material for: Maternal Prenatal Infections and Biliary Atresia in Offspring
Source: JAMA Netw Open. 2024 Jan 3;7(1):e2350044. doi: 10.1001/jamanetworkopen.2023.50044 (PMC10765264; doi:10.1001/jamanetworkopen.2023.50044)
Supplement: Supplement 2. — Data Sharing Statement [file jamanetwopen-e2350044-s002.pdf]

Data Sharing Statement

Wang. Maternal Prenatal Infections and Biliary Atresia in Offspring. *JAMA Netw Open*.
Published January 03, 2024. doi:10.1001/jamanetworkopen.2023.50044

Data
Data available: No

Additional Information
Explanation for why data not available: Data is available from the Health and Welfare Data Center published by the Ministry of Health and Welfare. Due to legal restrictions imposed by the government of Taiwan in relation to the “Personal Information Protection Act”, data cannot be made publicly available. Requests for data can be sent as a formal proposal to the Health and Welfare Data Center Administration (https://dep.mohw.gov.tw/DOS/cp-5119-59201-113.html).
